# Clinical and Pathological Response to Neoadjuvant Anthracycline Based Chemotherapy in women with breast cancer

**DOI:** 10.4021/wjon223w

**Published:** 2010-08-29

**Authors:** Gharbi Olfa, Trabelsi Amel, Chafai Rim, Zayen Aymen, Ezzairi Faten, Hochlef Makrem, Ben Fatma Leila, Landolsi Amel, Khairi Hedi, Mokni Moncef, Ben Ahmed Slim

**Affiliations:** a Department of Medical Oncology, Farhat Hahed Hospital, Sousse, Tunisia; b Department of Pathology, Farhat Hahed Hospital, Sousse, Tunisia

**Keywords:** Breast cancer, Chemotherapy, Histology, Predictive factors

## Abstract

**Background:**

Neoadjuvant chemotherapy has been used as a primary treatment for locally advanced or inflammatory breast cancer, and recently extended to operable breast cancer. The aim of this study was to evaluate the predictive value of different histologic factors in breast cancer treated with neoadjuvant anthracycline chemotherapy in Tunisian women.

**Methods:**

A total of 109 stage II and III breast cancer patients who received neoadjuvant anthracycline chemotherapy were enrolled in this study. Using pretreatment biopsy materials, histologic grade was recorded and immunohistochemical studies were performed for estrogen receptor, progesterone receptor and Her2neu. We analyzed the associations among this histologic factors and clinical and pathological complete response. Statistical analysis used is SEM logiciel.

**Results:**

The overall clinical response was 63% (clinical partial response in 49% of cases and clinical complete response in 14% of cases). The pCR was 7%; in univariate analysis, clinical response rate by each factors were as follows: 63% in ER-positive tumors, 84% in ER-negative (P = 0.2), 59% in PgR-positive, 62% in PgR-negative (P = 0.3), 64% in HER2-positive, 62% in HER2-negative (P = 0.6), 60% in tumors of low nuclear grade and 63% in ones of high nuclear grade (P = 0.9).

**Conclusions:**

Biological markers that reliably predict clinical and pathological response to primary systemic therapy may have considerable clinical potential. The future of neoadjuvant therapy lies in tailoring treatment to individual patients by identifying response predictors.

## Introduction

Neoadjuvant chemotherapy was originally used as a standard treatment for inflammatory and locally advanced breast cancer, however, the combination of image-guided-core needle biopsy and new breast imaging modalities have substantially increased the numbers of women with operable breast cancers which can be treated with primary chemotherapy [1-3]. Neoadjuvant chemotherapy for locally advanced breast cancer has become a model for testing novel therapeutic regimen, as pathologic complete response (pCR) in an excellent intermediate end point to test effectiveness of therapy [2]. It is important to identify accurately histopathologic factors that may predict the response to neoadjuvant therapy. These factors should identify patients who will benefit mostly from treatment, and would permit a tailored approach in selecting the initial therapy that may yield the best clinical and pathological response and subsequent overall survival [4]. This study was designed to evaluate predictive values of histopathologic markers in clinical and pathologic response of breast cancers treated with neoadjuvant anthracycline based chemotherapy (NAC).

## Patients and Methods

This was a retrospective study of 109 patients with primary breast cancer who were treated in our Hospital between January 2006 and March 2009. Inclusion criteria for this study were patients with cancer in untreated stages II and III (T1-T4, N0-2, M0) with invasive primary breast cancer. All patients had unresected disease and were considered candidates for primary chemotherapy. Pretreatment core biopsy histologic features including tumor type and histologic grade (modified Scarff-Bloom-Richardson SBR grades 1-3) was recorded. Immunohistochemical stains for estrogen receptor (ER), progesterone receptor (PR), and Her-2/neu were obtained on the initial core biopsy. HER2 positivity is defined as 3+ markedly positive in more than 30% of tumor cells. All patients received anthracycline-based chemotherapy regimen with 5-fluorouracil, adriamycin or epirubicin, and cyclophosphamide (FAC/FEC) every 3 weeks during 3 - 6 cycles before surgery. Tumor measurements were performed by physical examination at the baseline and after final cycle of neoadjuvant therapy. Clinical response to chemotherapy in tumor and in nodes was noted according to RECIST criteria [5]: complete response (CR) was defined as no residuel palpable abnormality; partial response (PR) as greater than 50% tumor shrinkage; stable disease (SD) as a decrease of less than 50% or an increase of less than 25%; progressive disease (PD) as an increase of at least 25% or the appearance of new lesions. The pathologic response in the surgical excision specimen was divided into complete response (cPR): no residual invasive tumor identified either in the breast or the lymph nodes, partial response (pPR): small foci of residual tumor, and no response: no change in tumor size with no treatment effect identified. We also used LeChevalier criteria for classification of pathologic response. We evaluated the relationship between the response rate to neoadjuvant chemotherapy and hormonal receptor, HER2 status, and nuclear grades. Statistical analysis was performed by SEM statistical package; the effect of biological factors on the response rate to neoadjuvant chemotherapy was assessed by the Chi-square test.

## Results

Median age was 47 years (range 28 to 78 years), median size of initial tumor was 6 cm (range 1 to 17cm), TNM distribution was: 36% T3, 30% T4b, 16% T4d, 8% T4c, 5% T2, 5% T4a, 22% of tumors were N2, 36% N1, 42% N0.

SBR grades were: 60% grade 2, 18% grade 1, 18% grade 3. Her2Neu was 3+ in 26%, hormonal receptors were positive in 70% of cases (positive ER in 70% of cases, positive PR in 60% of cases). The principle clinico-pathologic features were reported in table I. All patients received neoadjuvant chemotherapy with anthracyclines: 94% FEC100 (doxorubicin, 5 Fluouracil, cyclophosphamid), median number of cycles was 4 (extremes 2 to 8), median delay from last cycle of chemotherapy and surgery was 36 days. The surgery was radical in 98% of cases, median number of nodes in axillary lymph node dissection was 13 (range 5 to 29). Clinical response in breast according to RECIST criteria was: PR in 65% of cases, CR in 14% of cases, SD in 21% of cases, PD in 2% of cases. Clinical response in nodes according to RECIST criteria was: CR in 64% of cases, PR in 28% of cases, SD in 4.5% of cases, PD in 3% of cases. Pathologic response in the surgical excision specimen was complete response (cPR) in 7% of cases, partial response (pPR) in 68% of cases, and no response in 25% of cases. Distribution of responses by LeChevalier criteria was: grade 3 in 65% of cases, grade 4 in 28% of cases, grade 1 in 7% of cases. Seventy-eight percent of patients were N+ after neoadjuvant chemotherapy and median number of positive nodes was 3 (range 0 to 18). Clinical response rate by each factors were as follows: 61% in ER positive tumors, 84% in ER negative (P = 0.20), 59% in PgR positive, 62 % in PgR negative (P = 0.30), 64% in HER2 positive, 62% in HER2 negative (P = 0.6), 60% in tumors of low nuclear grade and 63% in ones of high nuclear grade (P = 0.9) (Table 2). Pathologic response rates by each factor were as follows: 42% in RH positive tumors, 57% in RH negative (P = 0.71), 60% in HER2 positive, 55% in HER2 negative (P = 0.71), 14% in tumors of low nuclear grade, and 85% in ones of high nuclear grade (P = 0.88).

**Table 1:** Clinico-pathological Features

Characteristics		Number of Patients	Percentage
Age (years)	< 50 years	62	56%
	≥ 50 years	47	43%
Menopausal status	Pre-	46	42%
	Post	63	58%
Clinical T stage*	T2	6	5.5%
	T3	39	36%
Clinical node stage*	N0	46	42%
	N1	39	36%
	N2	24	22%
Histology	Invasive ductal carcinoma	102	93%
	Invasive lobular carcinoma	6	5.5%
	Other	1	0.9%
Histological grade	Grade 1	20	18%
	Grade 2	64	59%
	Grade 3	20	18.5%
ER	Positive	73	70%
	Negative	32	30%
PR	positive	44	42%
	negative	61	58%
HR	positive	67	70%
	negative	29	30%
HER2	0/+/++	58	53%
	+++	28	26%

* TNM classification: ER - estrogen receptor, PR - progesterone receptor, HR - hormonal receptor

**Table 2:** Univariate Analysis of Clinical Response Rates by Histopathological Factors

Parameters		N° of Patients	N° With cCR (%)	P Value
Histological grade	Grade I, II	20	12 (60%)	0.9
	Grade III	84	53 (63%)	
pCR	No	100	6 (61%)	0.2
	Yes	8	7 (87%)	
ER	Positive	73	45 (61%)	0.2
	Negative	32	19 (84%)	
PR	Positive	44	26 (59%)	0.3
	Negative	61	38 (62%)	
HER2	0/+/++	58	36 (62%)	0.6
	+++	28	18 (64%)	

## Discussion

Breast cancer is the most frequent malignancy in women in the world and in Tunisia, it accounts for 20-25% of malignant tumors in women with annual incidence of about 800 - 1000 cases. The particularities of Tunisian women with breast cancer were the high rate of this cancer among women younger than 35 years (11%), and the alarming clinical profile (40.2% of cases have a tumor with a clinical diameter equal or greater than 5 cm) [6].

Neoadjuvant chemotherapy is the standard of care for locally advanced breast cancer and is used increasingly for large operable breast cancer. It shows better results in terms of the rate of response to treatment and a reduction in the requirement of mastectomy [1-3]. Alvarado et al [1] reported in a study of 205 patients that a reduction in tumor size occurred in 40% of patients, 12% had a cCR, and 28% a cPR, only 8% had no histologic evidence of residual invasive primary breast carcinoma or axillary metastases after 4 cycles of NAC (pCR), this result is comparable to our study. A reduction in tumor size occurred in 50% of our patients. The percentage of patients in this study who had a cCR (13%) was similar to that noted in other studies using only anthracycline based chemotherapy (range 12-30%) [2, 3]. In a neoadjuvant setting, the combination of taxanes and anthracycline is among the most actively described chemotherapy regimen. Bear et al [6] reported that the addition of docetaxel increased the overall clinical response rate, and this finding has also been supported by other studies that cited improved tumor regression with the use of docetaxel in the neoadjuvant setting [7, 8]. The pCR rates by neoadjuvant chemotherapy reported in the past were around 30% or less [9-12]; if we perform neoadjuvant chemotherapy only for cases likely to benefit, we could obtain a high pCR rate and avoid the undesirable side effects in other cases. Neoadjuvant chemotherapy provides an excellent model for evaluation of potential predictive factors. Information on the differential histologic response of primary breast tumors and axillary metastases to NAC is limited [4].

Furthermore, the clinical response to NAC does not always accurately reflect the pathologic response. On the other hand, there are different systems for assessing pathologic responses, and this leads to difficulties in comparing results across studies [1, 9]. The definition of pCR remains variable, and there is a lack of general consensus. We have included patients with complete disappearance of tumor in the breast as well as in the axillary lymph nodes in our definition of pCR. In the present series, 7% of the patients had a pCR. There is a paucity of published data concerning the incidence and outcome of patients with a pCR in the primary tumor and axillary lymph nodes after NAC. Most of the literature on NAC concerning pCR rates refers to and reports on pCRs in the primary tumor alone. Kuerer et al [10] did not find evidence of invasive tumor in the breast primary and axillary lymph nodes after NAC in 12% of their patients. On the other hand, Fisher et al [11] found a 7% rate of pCR in the primary tumor and axillary lymph nodes in 185 patients with local operable breast cancer and clinically positive axillary lymph nodes.

In the majority of studies [1, 3, 12], histological grade assessed from available pretreatment core biopsy was found to be correlated significantly with the type of pathologic response. Tumors with a poor nuclear grade were more likely to have a pCR than tumors with a low nuclear grade. Wang et al [13] reported that nuclear grade, evaluated according to the modified Black nuclear grading system, and mitotic activity correlated significantly with the histological therapeutic effect of neoadjuvant fluouracil, doxorubicin and cyclophosphamide (FAC) therapy. There are data that suggest that steroid receptor negativity predicts chemosensitivity. Colleoni et al [14] have demonstrated, like in our study, that tumors negative for estrogen or progesteron receptors demonstrated clinical and pCR rates superior to that of ER-positive tumors. Several other studies have also demonstrated a statistically greater response rate to NAC in steroid-receptor-negative patients [15], the underlying mechanism by which lack of ER-sensitive cells to apoptosis by chemotherapy is not fully established, but in vitro studies suggest that ER signaling can increase levels of bcl-2 and induce anthracycline resistance [16]. Kuerer et al [10] reported, like in our study, that ER-negative patients obtained a higher pCR rate than ER-positive patients with doxorubicin-containing neoadjuvant chemotherapy.

Data in the neoadjuvant setting are conflicting in the impact of HER-2 on response to NAC. Estevez et al [17] found no association between overexpression of HER-2 and the clinical and pathological response with weekly docetaxel as neoadjuvant chemotherapy for stage II and III breast cancer. Learn et al [18] proved that the addition of docetaxel to anthracycline-based chemotherapy improved clinical response rate in HER-2 negative breast cancer patients. Four randomized studies in the adjuvant setting showed that HER2-positive patients obtained more benefit from anthracycline-containing chemotherapy than HER2-negative patients [19]. In contrast, Zhou B et al [4] reported that overexpression of HER-2 and negative hormonal receptor status are much more likely to respond to neoadjuvant chemotherapy taxane and anthracycline chemotherapy than those with the opposite characteristics; in their multivariate analysis, overexpression of HER-2, remained as an independent variable in predicting the cCR and negative ER, was the only parameter that maintained statistical significance in predicting the pCR. It is clear that the impact of HER-2 on response to NAC should be tested in a great number of prospective trials to make a final conclusion. Ki-67 nuclear antigen served as predictive marker is controversial in the majority of studies [9-13]. Clahsen et al [20], in a study of 441 patients, reported that p53-negative cases obtained significantly more benefit from chemotherapy than p-53 positive cases, and the mechanism of resistance to doxorubicin therapy in breast cancer was related to specific p53 gene mutations. The histological category of chemo-sensitive carcinoma is a significant predictive factor for the efficacy of chemotherapy. The prediction of the efficacy of chemotherapy can properly select good candidates who will respond well to the treatment and also can exclude poor candidates who will have undesirable side effects instead of the benefits by the treatment [21].

In conclusion, biological markers that reliably predict clinical and pathological response to primary systemic therapy may have considerable clinical potential. The future of neoadjuvant therapy lies in tailoring treatment to individual patients by identifying response predictors.
